# HBx-Associated Reactivation of the IGF2 Locus in Chronic HBV Infection and HBV-Related Hepatocarcinogenesis: Evidence Boundaries and Biomarker Implications

**DOI:** 10.3390/biomedicines14071440

**Published:** 2026-06-25

**Authors:** Xiaojuan Wu, Jinghong Liu

**Affiliations:** 1 Department of Gastroenterology, Ganzhou Hospital-Nanfang Hospital, Southern Medical University (Ganzhou People’s Hospital), Ganzhou 341000, China; 2 Department of Gastroenterology, The First Affiliated Hospital of Jinan University, Guangzhou 510632, China; jhliu427@163.com

**Keywords:** HBV, HBx, IGF2, IGF2/H19, cccDNA, HBV DNA integration, epigenetic remodeling, hepatocarcinogenesis, hepatocellular carcinoma, biomarker development

## Abstract

Chronic hepatitis B virus (HBV) infection remains one of the main causes of hepatocellular carcinoma (HCC), even though vaccination and long-term viral suppression have reduced new infections and circulating viral replication. This residual cancer risk suggests that serum HBV DNA alone does not capture the full biology of HBV-related carcinogenesis. Hepatitis B virus X protein (HBx) is a relevant entry point because it maintains the transcriptional competence of covalently closed circular DNA (cccDNA), engages host chromatin regulators, and may persist in tumors as cccDNA-derived, integration-derived, full-length, truncated, or fusion forms. This review focuses on a specific question: does the available literature support HBx-associated reactivation of the IGF2 locus in chronic HBV infection and HBV-related hepatocarcinogenesis, and, if so, at which regulatory layer is the claim defensible? The most direct evidence remains promoter-proximal. Classic mechanistic work shows acute HBx-dependent activation of IGF2 promoter P4 through Sp1- and PKC/ERK-dependent signaling. Human tissue and cell-based studies also support a broader fetal-promoter compartment, including P3/P4 transcript enrichment, local promoter hypomethylation, MBD2-HBx-CBP/p300 recruitment, and increased histone H3/H4 acetylation. These observations do not, however, establish HBV exclusivity, uniform loss of imprinting, or direct HBx-mediated rewiring of the human IGF2/H19 topological domain. Recent integration-aware and long-read studies further argue against treating tumor-stage HBx as a single biological variable. In the present evidence framework, HBx-associated IGF2 locus reactivation is therefore more appropriately viewed as a stage-aware, promoter-resolved, biomarker-oriented hypothesis than as a universal mechanism or a treatment algorithm for HBV-related HCC.

## 1. Introduction

Chronic HBV infection remains a major cause of cirrhosis and HCC, despite the protection provided by vaccination and the ability of nucleos(t)ide analogs to suppress viral replication [1,2,3]. The persistence of HCC risk after prolonged viral suppression is not a paradox; it reflects the fact that circulating HBV DNA is only one part of a larger carcinogenic process. Intrahepatic viral templates, ongoing viral transcription, HBV DNA integration, and durable virus-associated epigenetic disturbance can remain relevant as chronically injured liver progresses toward malignant transformation.

Among HBV gene products, HBx is the viral factor most plausibly positioned between persistent infection and host transcriptional change. In infection-relevant systems, HBx is required to initiate and maintain productive replication by preserving the transcriptional competence of cccDNA [4]. The best-defined mechanism involves DDB1-containing CUL4 ubiquitin ligase machinery and degradation of the SMC5/6 restriction complex, which releases repression of episomal HBV DNA [5,6]. Broader reviews of HBx biology and HBV-associated epigenetic disruption support this restricted framing, although they are not IGF2-specific evidence [7,8]. Studies of cccDNA chromatin further connect HBx with histone acetylation balance, SETDB1-associated repression, PRMT1 interaction, HIRA-dependent cccDNA chromatin establishment, and chromatin–reader interactions [9,10,11,12,13]. Taken together, these studies justify describing HBx as a chromatin-active viral regulator. They do not, on their own, prove reprogramming of a host locus.

The IGF2/H19 locus is useful precisely because it forces the argument to be promoter-resolved. IGF2 is an oncofetal growth-factor gene located in the imprinted 11p15.5 IGF2/H19 domain. Its expression depends on promoter use, parental-origin control, local DNA methylation, histone marks, enhancer access, and higher-order chromatin organization. Adult human liver is mainly P1-driven, whereas fetal liver preferentially uses P2–P4 promoters [14,15,16,17,18]. The relevant issue is therefore not simply whether total IGF2 rises in HBV-related HCC. The sharper question is whether HBx is associated with a shift toward a fetal-promoter-dominant and epigenetically permissive IGF2 locus state.

### 1.1. Scientific Question and Scope

The review is built around a narrow question: does the literature support HBx-associated reactivation of the IGF2 locus during chronic HBV infection and HBV-related hepatocarcinogenesis, and where does that claim remain evidence-based? Current data favor a local, promoter-proximal model, not a domain-wide causal model. HBx can acutely prime IGF2 promoter P4 through Sp1- and PKC/ERK-dependent mechanisms [19,20]. Human tissue and cell-based studies extend the argument to a P3/P4 fetal-promoter compartment associated with local hypomethylation, MBD2-HBx-CBP/p300 recruitment, and increased H3/H4 acetylation [21,22,23]. Direct HBx-mediated rewiring of the human IGF2/H19 topological domain has not been demonstrated.

### 1.2. What This Review Adds

- The review uses the IGF2/H19 locus as a promoter-resolved host-locus model, rather than treating IGF2 as another pathway item in a broad HBx catalog.
- It keeps P4 transactivation, P3/P4 methylation, promoter switching, loss of imprinting, and IGF2/H19 domain topology as separate evidentiary layers.
- It develops HBx-associated IGF2 reactivation as a stage-aware and source-aware biomarker-development hypothesis, not as a universal HBV-HCC mechanism or an immediate treatment algorithm.

## 2. Methods and Search Strategy

This article is a structured narrative review, not a formal systematic review. We searched PubMed, Web of Science Core Collection, and Scopus and then used backward and forward citation tracking around key primary studies. The last search date was 22 April 2026. The primary search string was: ((HBV OR “hepatitis B virus” OR HBx OR “hepatitis B virus X protein”) AND (IGF2 OR “IGF-II” OR “IGF2/H19” OR H19 OR imprinting OR methylation OR promoter OR P3 OR P4 OR CTCF OR enhancer OR chromatin)) AND (hepatocellular carcinoma OR HCC OR hepatocarcinogenesis). Additional searches were run for HBx source biology and clinical context: (“HBV DNA integration” OR integrant OR truncated HBx OR fusion HBx OR long-read OR transcriptome) AND (HCC OR liver); (HBV AND guideline) OR (hepatocellular carcinoma AND guideline) OR (IMbrave150 OR HIMALAYA OR CheckMate 9DW OR CARES-310).

Eligible sources included original mechanistic studies of HBx and IGF2 regulation, human tissue studies with promoter-resolved, methylation-resolved, chromatin-resolved, or integration-aware data, infection-competent or endogenous-expression HBV models, major society guidelines, and pivotal phase III HCC trials relevant to biomarker interpretation. We excluded non-peer-reviewed claims, broad HBx pathway catalogs without direct IGF2 relevance, studies that could not be interpreted at the locus level, and therapy discussions not tied to biomarker development. Evidence was weighted in the following order: human tissue > infection-competent or endogenous-expression systems > integration-aware studies > stable/transient HBx overexpression > promoter-reporter assays. Because the field is mechanistically heterogeneous and this review was not prospectively registered, the synthesis is presented as critical narrative work rather than as a systematic review. Table 1 gives the search and evidence-weighting framework.

Because this article was designed as a critical narrative review rather than a systematic review, the search was not intended to generate an exhaustive census of all eligible records, a PRISMA flow diagram, or duplicate independent screening. The search strategy was used to identify and cross-check primary mechanistic, human tissue, integration-aware, and translational studies that directly informed the HBx-IGF2 question. Candidate sources were retained when they contributed to at least one predefined interpretive layer: HBx source and form, IGF2 promoter usage, P3/P4 methylation, loss of imprinting, chromatin recruitment, IGF2/H19 topology, HBV integration, or biomarker interpretation in HCC. Studies were not used as primary evidence when they addressed broad HBx pathway activity without IGF2 locus resolution, lacked promoter- or locus-level interpretability, or extrapolated therapeutic implications without biomarker relevance. Therefore, Table 1 should be read as a narrative source-selection and interpretive framework, not as a formal systematic evidence-grading scheme.

## 3. Human IGF2/H19 Locus: Promoter and Regulatory Definitions

The human IGF2 gene is classically described as having four promoters, P1 through P4. They generate transcripts with distinct 5′ leader exons while encoding the same IGF-II protein [14,15]. Fetal human liver preferentially uses P2–P4, whereas postnatal liver shifts toward a P1-dominant adult pattern [16]. The shift is not an all-or-none switch: all four promoters can be detected after birth, P4 may remain measurable in adults, and residual P3 transcription has been reported in some adult liver samples [16]. For that reason, fetal-promoter reactivation in HCC is better understood as disturbance of a regulated adult equilibrium, not as the reopening of a completely silent program.

Promoter switching, loss of imprinting (LOI), local hypomethylation, and domain topology often appear together in discussions of IGF2, but they are not equivalent. In human liver, P1 transcripts are generally biallelic, whereas P2–P4 retain parent-of-origin restriction during development [17,18]. LOI can occur in liver cancer. It should not be used as a synonym for promoter switching. A tumor may reactivate P3/P4 without complete biallelic IGF2 expression, may relax imprinting unevenly across promoters, or may show local promoter hypomethylation without proven domain-scale rewiring.

Species context matters here. The adult human P1-dominant program is not fully mirrored in standard rodent systems, although a P1-homologous transcript has been described in baboon liver [24]. Rodent models remain valuable for testing IGF-II biology, but they are poor surrogates for the adult human promoter hierarchy. This caveat becomes important when HBx and IGF2 findings from hepatoma lines or mouse models are used to explain adult human HBV-related HCC.

The H19 imprinting control region (ICR)-CTCF model is still a useful framework for enhancer insulation, but it cannot be treated as a complete predictor of human tissue behavior. Foundational studies established methylation-sensitive CTCF binding and parent-specific chromatin loops at the H19/Igf2 locus [25,26,27]. Human tissue data are more complicated: CTCF occupancy at the IGF2/H19 ICR is insufficient to predict IGF2/H19 output [28]. Recent syntheses place promoter selection, multiple differentially methylated regions, transcription-factor inputs, enhancer logic, and transcript-level controls into the same regulatory picture [29]. In this review, local P3/P4 methylation and promoter activity are treated as supported HBx-associated layers; human 11p15.5 topology remains plausible but unproven unless it has been directly tested.

**Figure 1:**
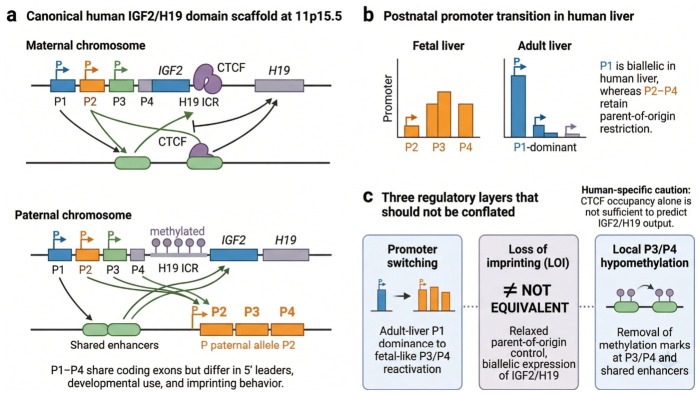
Developmental organization of the human IGF2/H19 domain and regulatory layers relevant to HBx interpretation. The figure distinguishes adult-liver P1 dominance, fetal P2–P4 usage, promoter switching, LOI, and local P3/P4 hypomethylation. These related layers should not be cited interchangeably as evidence for domain-wide IGF2/H19 rewiring. Abbreviations: HBV, hepatitis B virus; HBx, hepatitis B virus X protein; IGF2, insulin-like growth factor 2; LOI, loss of imprinting; ICR, imprinting control region; CTCF, CCCTC-binding factor.

Figure 1 and Table 2 define the regulatory layers that need to remain separate when HBx-associated IGF2 effects are interpreted.

## 4. HBx Biology Relevant to Host-Locus Interpretation

### 4.1. HBx, cccDNA Transcription, and the DDB1-CUL4-SMC5/6 Axis

The viral life cycle should anchor any discussion of HBx-associated host-locus remodeling. After viral entry and rcDNA conversion, cccDNA persists as a chromatinized minichromosome and supplies the template for viral RNAs. HBx is required for productive transcription from this template in natural infection models, including primary human hepatocytes and differentiated HepaRG systems [4]. The strongest mechanistic explanation is SMC5/6 antagonism: HBx binds DDB1, recruits CUL4 ubiquitin ligase machinery, and promotes SMC5/6 degradation, thereby relieving repression of extrachromosomal viral DNA [5,6].

This mechanism restricts the interpretation of HBx rather than expanding it indefinitely. Older work often described HBx as a general transactivator of many cellular promoters. The DDB1-CUL4-SMC5/6 axis provides a defined viral mechanism for derepressing chromatinized HBV episomes. It does not demonstrate direct IGF2 regulation. It does, however, make promoter-level and chromatin-level host-locus effects plausible in chronically infected hepatocytes in which HBx is repeatedly produced.

Several cccDNA studies support this cautious framing. Nuclear HBx can associate with the HBV minichromosome and modify the recruitment of histone acetyltransferases, deacetylases, and cccDNA-associated histone acetylation [9]. Other studies connect HBx with relief of SETDB1-associated repression, interaction with PRMT1, HIRA-supported cccDNA chromatin establishment, and Spindlin1-associated cccDNA transcription [10,11,12,13]. These studies should not be cited as direct proof of IGF2 regulation. Their more defensible role is to establish that HBx operates as a chromatin-active viral protein in infection-relevant systems. The evolutionary conservation of HBx-mediated SMC5/6 antagonism across mammalian hepadnaviruses further underlines this function [30]. Pharmacologic disruption of HBx-DDB1 has been explored to suppress cccDNA transcription [31], and HBx-induced SMC5/6 degradation may also compromise homologous recombination-mediated DNA repair [32].

### 4.2. HBx Source and Form Across Disease Stages

HBx should not be treated as a single stable variable across HBV-related liver disease. In chronically infected hepatocytes, the clearest biological anchor is cccDNA transcriptional competence. In established HCC, HBV DNA integration becomes increasingly important and can generate persistent viral transcripts, including transcripts containing HBx sequence [33,34,35,36,37]. Integrated HBV DNA is not merely a historical scar of infection. It can change host genomic structure, activate cancer-related loci, produce viral–host chimeric transcripts, and obscure the origin of viral RNA [33,34,35,36,37].

**Figure 2:**
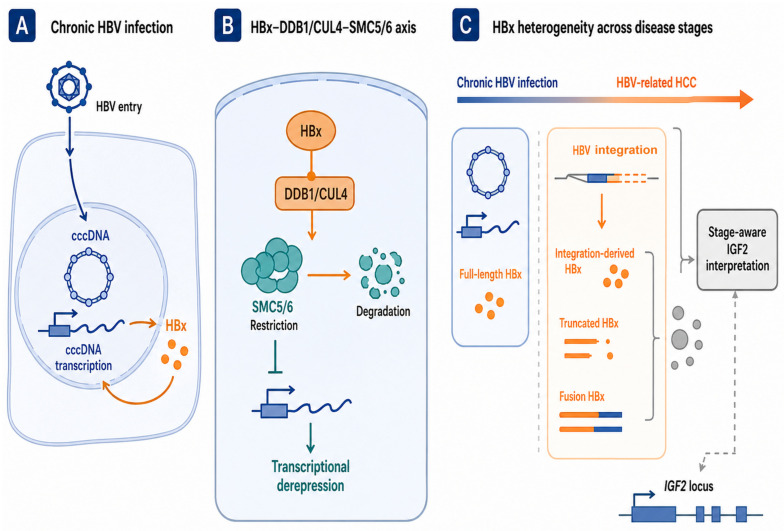
HBx viral-stage biology relevant to IGF2 locus interpretation. HBx supports cccDNA transcription by antagonizing SMC5/6 through DDB1-CUL4-associated mechanisms. During progression from chronic HBV infection to HBV-related HCC, HBx may arise from cccDNA-derived, integration-derived, full-length, truncated, or fusion species. This stage-aware and source-aware framing limits how far HBx-associated IGF2 locus reactivation can be generalized.

HBx protein form adds another layer of uncertainty. Full-length HBx, C-terminally truncated HBx, and fusion HBx species may differ in localization, transactivation potential, binding partners, and oncogenic behavior. C-terminally truncated HBx has been associated with metastasis and increased invasiveness in HBV-related HCC, including c-Jun/MMP10 activation and metabolic reprogramming [38,39]. Fusion HBx from HBV integrants has been linked to deregulated endoplasmic reticulum stress responses and tumor-promoting behavior [40]. Most IGF2 studies did not distinguish cccDNA-derived from integration-derived HBx, or full-length from truncated/fusion HBx. That omission is a real interpretive limitation, not a minor annotation issue. Figure 2 and Table 3 summarize these source and form distinctions across disease stages.

## 5. From Chronic Liver Disease to HBV-Related HCC: What Is HBV-Specific?

Oncofetal IGF2 re-expression was described before the HBx mechanistic literature took shape. Early mouse and human studies detected IGF-II re-emergence during hepatocarcinogenesis and in subsets of HCC, cirrhosis, dysplastic nodules, and chronic HBV-associated liver disease [41,42,43,44,45]. Later promoter-resolved studies showed that increased IGF2 expression in HCC is often transcriptional and linked to fetal-promoter use, rather than simply reflecting total-gene upregulation [45].

The first boundary is etiologic. Fetal IGF2 promoter reactivation is not unique to HBV-related disease. HCV-related chronic hepatitis, cirrhosis, and HCC also show fetal P3/P4 activation, and IGF2 hypomethylation in HCV cirrhosis has been associated with later HCC occurrence [46,47]. HBx should therefore not be presented as the sole trigger of fetal IGF2 promoter activity. A more defensible HBV-specific question is whether HBx provides a promoter-resolved and epigenetically stabilizing route that enriches or maintains this state in a subset of HBV-related HCC.

Human HCC studies fit this narrower interpretation. Loss of P1-dominant adult promoter usage is frequent, whereas total IGF2 overexpression and LOI occur only in subsets [48,49,50]. In HBV-associated tumors, microarray and promoter-usage studies linked H19/IGF2 deregulation to reduced P1 contribution and reactivation of P3/P4 in tumors and adjacent nontumorous liver [51,52]. Fetal-promoter transcripts were associated with poorer differentiation, p53 mutation, and portal-vein tumor embolus in HBV-related cohorts [52]. These findings support a P3/P4 fetal-promoter compartment model. Within that model, P4 carries the strongest direct HBx transactivation evidence, whereas P3 carries more tissue-level and prognostic weight.

## 6. Primary Evidence Linking HBx to IGF2 Promoter Regulation

A model-system caveat is necessary before interpreting the primary HBx-IGF2 evidence. Several influential studies used hepatoma reporter systems, HepG2, Huh7, Hep3B, stable HBx expression, transient HBx transfection, or promoter/methylation constructs. HepG2 is hepatoblastoma-derived and is therefore intrinsically closer to an oncofetal IGF2/11p15.5 regulatory state than normal adult hepatocytes. Adult HCC-derived lines such as Huh7 and Hep3B are also clonal, transformed systems and may not reproduce the nonclonal, P1-dominant promoter equilibrium of adult human hepatocytes. These systems are valuable for identifying promoter elements, signaling requirements, and chromatin associations, but they may overestimate the ease, durability, or clinical generalizability of HBx-associated IGF2 reactivation. The studies below are therefore interpreted as mechanistic or supportive evidence rather than definitive proof of endogenous adult-liver locus reprogramming.

Table 4 separates the main HBx-IGF2 evidence into direct promoter evidence, human association, model-supported inference, and contextual data.

### 6.1. P4-Sp1-PKC/ERK: Strongest Direct Promoter-Level Evidence

The clearest promoter-level evidence remains the P4-Sp1 model. Lee and colleagues showed that HBx enhances transcription from IGF2 promoter P4 through Sp1-responsive elements in hepatoma reporter systems [19]. In this setting, HBx did not function as a sequence-specific DNA-binding factor; it increased the ability of host transcriptional machinery to activate a developmentally restricted promoter. Kang-Park and colleagues then showed that PKC and p44/42 MAPK/ERK1/2 signaling are required for HBx-induced IGF2 promoter activation [20].

The inference from these studies should stay narrow. They support acute P4 promoter activation in reporter-based or hepatoma-cell contexts. They do not prove durable locus memory, uniform activation of all fetal promoters, complete LOI, or domain-wide IGF2/H19 reconfiguration. They also do not show that P4 is always the dominant fetal promoter in clinical HBV-related HCC. Their value is still considerable: they provide the most direct mechanistic anchor for an HBx-IGF2 model.

HBx-mediated IGF2 promoter activation is likely context-dependent and may cooperate with other oncogenic pressures. In combined HBV–aflatoxin settings, the codon-249 p53 mutant can enhance IGF2 transcription largely through P4 by increasing Sp1- and TBP-containing transcription complexes, and HBV-related HCC tissue studies have linked fetal-promoter reactivation with p53 mutation [52,56]. HBx is therefore better described as a viral amplifier or organizer within a wider oncogenic context than as the sole source of fetal IGF2 promoter reactivation.

### 6.2. P3/P4 Hypomethylation and Local Chromatin Stabilization

Human tissue studies link abnormal promoter use to local epigenetic change. Tang and colleagues reported that P4 promoter hypomethylation is more frequent in HCC than in normal liver and correlates with increased P4 transcripts, poorer differentiation, and portal-vein tumor thrombus [21]. Similar abnormalities in adjacent nontumor tissues favor a field-effect interpretation rather than a purely late tumor event.

Subsequent HBV-positive HCC work extended the model to P3. P3 transcript abundance was higher and P3 methylation lower in HBV-positive tumors than in HBV-negative tumors. P3 expression correlated positively with HBx, and P3 methylation correlated inversely with transcript level. Stable or transient HBx expression in cell systems increased P3 transcription and reduced P3 methylation [22]. These data support a P3/P4 compartment model, but P3 and P4 should not be assigned the same mechanistic status. P4 is the better-defined promoter for direct HBx transactivation; P3 is supported mainly by tissue association, methylation data, and clinical relevance.

**Figure 3:**
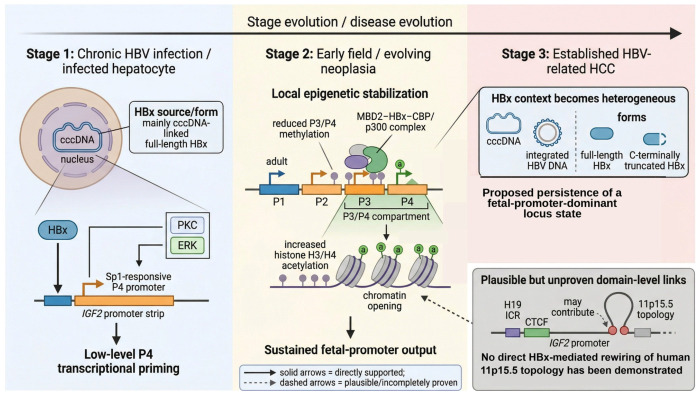
Stage-aware working model of HBx-associated fetal IGF2 promoter reactivation. Stage 1 depicts promoter-proximal P4 priming through Sp1- and PKC/ERK-dependent signaling. Stage 2 depicts local P3/P4 epigenetic stabilization through reduced promoter methylation, functionally implicated MBD2-HBx-CBP/p300 recruitment, and increased H3/H4 acetylation. Stage 3 highlights tumor-stage HBx heterogeneity and the absence of direct proof for HBx-mediated human 11p15.5 domain rewiring.

The most integrated local-chromatin model comes from Liu and colleagues. In HCC cells and tissues, HBx increased IGF-II expression, reduced methylation at P3 and P4, enhanced MBD2 expression, interacted with MBD2 and CBP/p300, increased recruitment of these factors to hypomethylated P3/P4 regions, and increased histone H3/H4 acetylation at the same promoters [23]. Knockdown experiments implicated endogenous MBD2 and CBP/p300 in full HBx-mediated IGF-II overexpression. A cautious conclusion is that HBx can participate in a chromatin-permissive state at local fetal IGF2 promoters. The causal order among demethylation, MBD2 recruitment, CBP/p300 engagement, histone acetylation, and transcript output remains incompletely resolved. Broader HBx-associated epigenetic perturbation at other host loci provides context but not direct IGF2-specific evidence [57,58]. Figure 3 summarizes the evidence-bounded working model.

## 7. Domain Architecture: Biologically Relevant but Not Proven to Be HBx-Directed

Promoter-level regulation is unlikely to explain the entire IGF2 phenotype because IGF2 lies within the IGF2/H19 domain. Domain architecture and enhancer–promoter contact can shape IGF2/H19 output, and HBV–host chromatin interaction studies show that HBV sequences can contact human chromatin in ways that may influence local gene regulation [53]. Recent HCC work showed that miR-483-5p can activate the IGF2/H19 enhancer, increase P2–P4 and H19 transcription, recruit p300 and RNA polymerase II, promote eRNA production, and induce enhancer–promoter looping through a MED1-associated mechanism [59].

These findings make long-range chromatin mechanisms relevant to HCC, but they do not demonstrate HBx-dependent remodeling of IGF2/H19 domain topology. Human tissue data also caution against assuming that CTCF occupancy alone predicts IGF2/H19 output [28]. The evidence can be separated into three levels: direct support for HBx-mediated P4 priming and local P3/P4 epigenetic remodeling; plausible interaction between local promoter changes and domain architecture; and, at present, no direct proof of HBx-mediated rewiring of human 11p15.5 topology. This boundary should remain visible throughout the manuscript.

## 8. Biological Consequences and Subset Logic

IGF2 should not be reduced to a generic dedifferentiation marker. IGF-II can signal through IGF1R and insulin receptor-A (IR-A), and IGF-axis activation defines a molecular subclass of HCC enriched for proliferation-related biology [54,55,60]. Tovar and colleagues linked IGF-axis activation in early HCC to fetal P3/P4 reactivation, IGF1R signaling, and proliferation-class features [54]. Martinez-Quetglas and colleagues later showed that IGF2 is epigenetically upregulated in a molecular subset of HCC and behaves as an actionable oncogene product in experimental systems [55].

HBx-relevant functional data are compatible with a subset model, although they do not resolve human promoter hierarchy. In mouse models, HBx-induced IGF-II contributes to hepatomegaly, abnormal hepatocyte growth, epithelial–mesenchymal transition, and SUMO-associated loss of E-cadherin [61]. Hepatocyte-specific Igf2 deletion can prevent DNA damage accumulation and tumor formation in experimental HCC settings [62]. These studies support biological activity. They do not justify a universal claim.

The most defensible biological interpretation is subset-based rather than universal. Fetal-promoter-dominant IGF2 expression may mark HBV-related HCC enriched for developmental reversion, IGF-axis signaling, invasion-related behavior, and poorer prognosis. It should not be presented as pan-HCC biology or as HBV-exclusive. Table 5 summarizes the level of support for the main mechanistic claims and indicates wording that should be avoided.

## 9. Translational Implications: A Biomarker-Development Framework Under Contemporary HCC Therapy

The translational relevance of the HBx-IGF2 axis has to be read against current HCC management, not against the older sorafenib-only era. Major guidance documents now place immune checkpoint inhibitor-based combinations at the center of first-line systemic therapy for advanced or unresectable HCC, while subsequent TKI or selected immunotherapy sequencing continues to evolve [63,64,65]. Older anti-IGF-axis trials explain why caution is necessary. Cixutumumab monotherapy produced no objective responses in unselected advanced HCC and caused substantial hyperglycemia; cixutumumab plus sorafenib showed limited efficacy and considerable toxicity in an unselected population [66,67]. These negative trials do not negate IGF2 biology. They show that histology-only enrollment is a weak clinical strategy for a pathway that appears subset-dependent.

The phase III treatment landscape has changed quickly. IMbrave150 established atezolizumab plus bevacizumab as a major first-line immunotherapy-based standard, HIMALAYA supported tremelimumab plus durvalumab, CARES-310 supported camrelizumab plus rivoceranib, and CheckMate 9DW supported nivolumab plus ipilimumab in appropriate unresectable HCC populations [68,69,70,71,72,73]. In this setting, a mechanistic pathway becomes clinically useful only if it defines a reproducible subset with prognostic, predictive, or enrichment value beyond established variables, including etiology, BCLC stage, AFP, liver function, vascular invasion, and treatment regimen. Current HBV guidance also reinforces a long-term viral and host-risk view of HCC risk, rather than reliance on serum HBV DNA alone [74].

The immune–microenvironmental relevance of IGF2 should also be interpreted cautiously. Emerging studies outside HBV-related HCC suggest that IGF2 or IGF-axis activity can be discussed in relation to metastatic behavior and tumor immune evasion [75], fibroblast-mediated T-cell exclusion and immunotherapy resistance [76], and macrophage/fibroblast chemokine programs that influence CD8+ T-cell recruitment [77]. These observations provide a rationale for considering IGF2 biology in the contemporary immune-checkpoint era, but they should not be treated as direct evidence that HBx-driven IGF2 promoter reactivation remodels the immune microenvironment in HBV-related HCC. For the present model, immune-context studies are therefore supportive translational background rather than primary HBx-IGF2 locus evidence.

The nearest clinical use of the HBx-IGF2 literature is therefore molecular stratification, not revival of an unselected anti-IGF strategy. Candidate readouts include promoter-resolved P3/P4:P1 transcript ratios, P3/P4 methylation, total IGF2/IGF-II abundance, HBx abundance, HBV integration status, and source-aware HBx classification. These assays should be tested first in retrospective, well-annotated HBV-related HCC cohorts and then in prospective biomarker cohorts within the current ICI/TKI era. At present, no IGF2 promoter-resolved assay should be used for clinical decision-making in HBV-related HCC outside research settings. Potential clinical intended-use scenarios are summarized in Table 6.

## 10. Limitations and Future Directions

### 10.1. Model-System Bias

The main methodological weakness in the HBx-IGF2 literature is model-system bias. Many mechanistic studies rely on HepG2, Huh7, stable HBx expression, transient HBx transfection, or promoter-reporter assays. HepG2 is hepatoblastoma-derived rather than a canonical adult HCC line, and hepatoblastoma itself is characterized by abnormal IGF2 regulation and 11p15.5 dysregulation [78,79,80]. A model already close to an oncofetal IGF2 state may make HBx-driven reactivation appear easier than it would be in adult human HBV-related HCC. This does not invalidate the studies. It does lower the confidence with which their results can be extrapolated. Even adult HCC lines, including Huh7 and Hep3B, should not be treated as neutral adult-liver surrogates because clonal transformation, long-term culture adaptation, and baseline fetal/oncofetal gene expression may distort the P1-to-P3/P4 promoter balance.

HBx experiments are also sensitive to expression level, cellular context, and assay design, as emphasized in technical standards for HBx research [81]. Future work should assign greater weight to infection-competent human liver organoids, primary human hepatocytes, differentiated HepaRG or NTCP-expressing systems, and well-characterized HBV-positive HCC models. Forced HBx overexpression remains useful for mechanism testing; it is weaker evidence for clinical causality.

### 10.2. Missing Source- and Isoform-Aware IGF2 Studies

Most IGF2 promoter and methylation studies did not specify whether HBx was cccDNA-derived, integration-derived, exogenous, full-length, C-terminally truncated, or fusion-derived. That missing information matters because these states can differ in template context, copy number, persistence, sequence, and protein partners. A stage-aware model requires promoter-resolved IGF2 measurements coupled to HBx source and isoform analysis. Long-read viral transcript sequencing, integration-aware DNA sequencing, and isoform-specific HBx assays should be paired with IGF2 promoter-resolved RNA sequencing and methylation profiling.

### 10.3. Experiments That Would Resolve the Key Uncertainties

A limited set of experiments would resolve much of the current ambiguity. A practical source-aware design would require paired viral-origin and host-locus measurements in the same specimens. In HBV-related HCC tissues, long-read viral transcriptome sequencing or integration-aware DNA/RNA sequencing should be paired with promoter-resolved IGF2 RNA-seq, targeted P3/P4:P1 transcript quantification, P3/P4 methylation profiling, and, where tissue permits, isoform-specific HBx immunoblotting or HBx-associated chromatin assays. This design would distinguish episomal from integrated viral transcripts, separate full-length, truncated, and fusion-derived HBx species, and test whether specific HBx sources or isoforms correspond to P4 activation, broader P3/P4 reactivation, local methylation change, or chromatin-permissive states. Such paired assays are more informative than measuring total IGF2 or total HBx in isolation. Promoter-resolved long-read RNA sequencing could determine whether HBx mainly affects P4, expands sequentially toward P3/P4, or produces a fetal-promoter program that varies by cell state. Phased methylome analysis could test whether P3/P4 methylation changes are allele-specific and whether they track with imprinting status. Chromatin assays, including H3/H4 acetylation mapping, CTCF/cohesin profiling, and chromosome-conformation capture, are needed to test whether local promoter changes propagate to 11p15.5 domain architecture. Integration-aware and isoform-specific HBx studies would clarify whether full-length, truncated, and fusion HBx species have distinct effects on the IGF2 locus. Prospective clinical cohorts should test whether a fetal-IGF2 locus state adds prognostic or predictive information beyond established clinical and molecular variables. Table 7 summarizes the experimental priorities.

## 11. Conclusions

The HBx-IGF2 literature supports a focused mechanistic synthesis, but not broad causal claims. The most direct evidence shows that HBx can acutely prime IGF2 promoter P4 through Sp1- and PKC/ERK-dependent signaling. Human tissue and cell-based studies support a broader P3/P4 fetal-promoter compartment associated with local promoter hypomethylation, functionally implicated MBD2-HBx-CBP/p300 recruitment, and increased histone H3/H4 acetylation. These findings justify a stage-aware model in which HBx contributes to local fetal-promoter reactivation and epigenetic stabilization at the IGF2 locus in a subset of HBV-related HCC.

The model has several limits that should remain explicit. Fetal IGF2 promoter reactivation is not HBV-exclusive; LOI is heterogeneous; human IGF2/H19 regulation cannot be reduced to murine ICR logic; and direct HBx-mediated rewiring of the human 11p15.5 domain has not been demonstrated. HBx source and form remain the most consequential unresolved variables. cccDNA-derived, integration-derived, full-length, truncated, and fusion HBx species may not exert the same pressure on the IGF2 locus. Translationally, this biology is best developed as a promoter-resolved, methylation-aware, integration-aware, and isoform-aware biomarker-enrichment framework rather than as a stand-alone therapeutic algorithm.

## Figures and Tables

**Table 1:** Narrative search strategy, source-selection criteria, and interpretive hierarchy. Abbreviations: HBV, hepatitis B virus; HBx, hepatitis B virus X protein; HCC, hepatocellular carcinoma; cccDNA, covalently closed circular DNA.

Component	Implementation in This Narrative Review	Reason for Inclusion
Databases and sources	PubMed, Web of Science Core Collection, Scopus, and backward/forward citation tracking of primary HBx, IGF2/H19, HBV integration, and HCC biomarker studies.	The field spans older promoter biology, contemporary HBV mechanistic work, integration-aware transcriptomics, and translational HCC literature.
Search window	Literature considered through 22 April 2026.	Allows inclusion of recent HBV integration and transcript-origin studies that affect interpretation of tumor-stage HBx.
Interpretive priority	Human tissue > infection-competent or endogenous-expression models > integration-aware studies > stable/transient HBx expression > promoter-reporter assays.	Prevents classic overexpression and reporter systems from being treated as sufficient evidence for durable tumor-state reprogramming.
Interpretive categories	Direct mechanistic evidence, human association, model-supported inference, contextual mechanistic plausibility, and unproven/hypothesis-generating claims.	Keeps mechanistic language proportional to the data.
Exclusions	Non-peer-reviewed claims, broad HBx pathway catalogs without IGF2 relevance, studies lacking locus-level interpretability, and therapy discussions not tied to biomarker development.	Maintains a virology-centered and locus-specific scope.

Note: This table does not represent a systematic review grading system. It specifies how source types were prioritized when interpreting mechanistic, human tissue, integration-aware, and translational evidence in this critical narrative synthesis.

**Table 2:** Regulatory layers at the IGF2/H19 locus that should not be conflated.

Layer	Operational Readout	What It Can Support	What It Cannot Support Without Additional Data
Promoter switching	Relative contribution of P1 versus P2–P4 transcripts, ideally by promoter-resolved qPCR, RNA-seq, or long-read transcriptomics.	Adult-to-fetal promoter reactivation and developmental reversion of IGF2 transcription.	Does not prove LOI, allelic relaxation, or topological remodeling.
Local promoter methylation	Bisulfite sequencing, methylation-specific PCR, or targeted methylation sequencing at P3/P4 regions.	Local epigenetic permissiveness linked to fetal-promoter output.	Does not by itself establish that HBx caused the change in human tumors.
LOI	Allele-specific expression using informative polymorphisms and parental-origin assignment.	Relaxation of parent-of-origin control.	Does not automatically identify the active promoter or prove P3/P4 activation.
Histone acetylation/chromatin recruitment	ChIP-qPCR/ChIP-seq/CUT&RUN for H3/H4 acetylation, CBP/p300, MBD2, HBx-associated recruitment.	Local chromatin-permissive state at promoter regions.	Does not prove long-range IGF2/H19 domain rewiring.
Domain topology	3C/4C/Capture-C/Hi-C plus CTCF/cohesin profiling, ideally allele-resolved.	Enhancer–promoter contact and insulation changes.	Should not be assigned to HBx without source-aware HBx perturbation and IGF2/H19-specific topology data.

**Table 3:** HBx source and form across HBV disease stages.

Disease Stage/Context	Likely HBx Source/Form	Relevance to IGF2 Locus Interpretation	Main Evidence Gap
Early chronic HBV infection	Mainly cccDNA-linked full-length HBx in infected hepatocytes.	Provides the most virologically grounded context for HBx-dependent transcriptional competence and possible early host-locus priming.	Direct promoter-resolved IGF2 assays in natural infection systems remain scarce.
Chronic inflammation and field change	Mixed cccDNA activity with accumulating integration events in long-lived hepatocyte populations.	May permit repeated viral and inflammatory pressure on the adult-to-fetal promoter equilibrium.	Most field-effect studies measure IGF2 but not HBx source or viral template origin.
Early neoplasia and HCC evolution	Integration-derived viral transcripts may become more prominent; full-length and truncated HBx may coexist.	Could help stabilize or select promoter states rather than initiate them de novo.	Few studies combine HBV integration mapping with promoter-resolved IGF2 transcriptomics.
Established HBV-related HCC	Integrated HBV DNA, C-terminally truncated HBx, fusion HBx, and residual cccDNA may contribute variably.	Tumor-stage IGF2 output may reflect heterogeneous HBx sources/forms rather than a single HBx mechanism.	No isoform-specific HBx-IGF2 chromatin study has been performed in clinical tissues.

**Table 4:** Primary evidence matrix for HBx-IGF2 promoter regulation.

Study/Year	System/Model	HBx Context	IGF2 Layer Tested	Main Finding	Key Limitation	Evidence Level	Citation
Lee et al., 1998	Hepatoma cell reporter systems	Exogenous full-length HBx	P4 transcription	HBx regulates Sp1-mediated activation of IGF2 promoter 4.	Reporter assay; not endogenous locus; no infection context.	Direct mechanistic for acute promoter priming	[19]
Kang-Park et al., 2001	Hepatoma cells with reporter/inhibitor assays	Exogenous HBx	P4 signaling dependence	HBx-induced IGF-II activation requires PKC and p44/42 MAPK/ERK1/2 signaling.	Reporter-dominant; no durable tumor-state inference.	Direct mechanistic for acute promoter priming	[20]
Tang et al., 2006	Human HBV-related HCC tissues	Endogenous HBV-associated context; HBx not directly manipulated	Promoter usage	HBV-related HCC shows abnormal fetal-promoter usage, supporting a P3/P4-oriented shift.	Association study; promoter use not directly assigned to HBx.	Human association	[52]
Tang et al., 2006	Human HCC tissues	Endogenous tumor context	P4 methylation and transcripts	P4 hypomethylation correlates with increased P4 expression and aggressive pathology.	HBx not directly tested; etiology-specific attribution limited.	Human association	[21]
Tang et al., 2015	HBV-positive vs. HBV-negative HCC specimens; HepG2-HBx/Huh7 transient systems	Tissue HBx plus stable/transient exogenous HBx	P3 transcripts and methylation	P3 expression is higher and P3 methylation lower in HBV-positive HCC; HBx correlates with P3 and reduces P3 methylation in cells.	Mixed tissue association and overexpression systems; methylation constructs are not equivalent to native locus context.	Human association plus supportive mechanistic inference	[22]
Liu et al., 2015	HCC cells and tissues	Stable HBx/endogenous HBV-associated context	P3/P4 methylation, MBD2-HBx-CBP/p300, H3/H4 acetylation	HBx promotes MBD2-HBx-CBP/p300 recruitment to hypomethylated P3/P4 and increases histone H3/H4 acetylation.	Causal order of demethylation–recruitment–acetylation remains unresolved; endogenous infection models lacking.	Direct locus-level mechanistic support, with caveats	[23]
Yang et al., 2020	3D HBV–host chromatin interaction mapping	Episomal and integrated HBV DNA	HBV–host chromatin topology context	HBV DNA can interact with human chromatin in 3D space, making domain-level host regulation plausible.	Not IGF2-specific; does not prove HBx-directed IGF2/H19 rewiring.	Contextual mechanistic	[53]
Qian et al., 2024	124 HCCs with WGS and Nanopore long reads	Integration-derived viral sequences	HBV integration structure/source heterogeneity	Complex HBV integrations reshape genomic architecture and complicate tumor-stage interpretation of HBx source/form.	Not IGF2-focused; source inference does not equal protein function.	Contextual mechanistic	[36]
Harris et al., 2025	Episomal/integrated HBV transcriptome mapping	Episomal vs. integrated transcripts	Transcript-origin heterogeneity	Episomal and integrated HBV transcriptomes are heterogeneous, reinforcing source-aware HBx interpretation.	Not coupled to HCC tissue IGF2 assays.	Contextual mechanistic	[37]
Tovar et al., 2010/Martinez-Quetglas et al., 2016	Human HCC subclasses and functional models	Not HBx-specific	IGF-axis subset biology	IGF-axis activation and IGF2 upregulation define biologically meaningful HCC subsets.	Not HBV/HBx-specific; does not define promoter hierarchy.	Translational subset support	[54,55]

**Table 5:** Mechanistic claim versus evidence level for the HBx-IGF2 working model.

Mechanistic Claim	Defensible Wording	Wording to Avoid	Evidence Level	Next Experiment Needed
HBx directly activates IGF2 promoter P4	HBx can acutely activate IGF2 promoter P4 in reporter/cell systems.	HBx reprograms the IGF2 locus in HCC through P4.	High for acute promoter priming	Endogenous HBx or infection-competent validation with endogenous IGF2 locus readouts.
HBx expands a broader fetal P3/P4 compartment	Human and cell-based studies support a broader P3/P4 fetal-promoter compartment associated with HBV/HBx.	HBx switches the adult-liver IGF2 locus to a fetal program.	Moderate	Promoter-resolved longitudinal and source-aware tissue studies.
HBx induces P3/P4 hypomethylation	HBx is associated with lower P3/P4 methylation in tissue/cell studies.	HBx is the cause of P3/P4 hypomethylation in patients.	Moderate	Allele-aware methylome analysis plus endogenous HBx perturbation.
HBx stabilizes local transcription via MBD2-HBx-CBP/p300	HBx can participate in a chromatin-permissive state involving MBD2-HBx-CBP/p300 recruitment and histone acetylation at P3/P4.	MBD2-HBx-CBP/p300 is the established mechanism of HBx-driven IGF2 overexpression in HBV-HCC.	Moderate	Independent replication, temporal causality, and infection-competent models.
HBx rewires human IGF2/H19 domain topology	Domain-level effects are biologically plausible but unproven for HBx at the human IGF2/H19 locus.	HBx disrupts the H19/ICR/CTCF architecture.	Low/unproven	Allele-resolved Hi-C/4C/Capture-C with CTCF/cohesin profiling in source-aware tissues.
Tumor-stage HBx behaves as a single biological variable	Tumor-stage HBx should be interpreted as source- and isoform-heterogeneous.	HBx in HCC as if it were one stable molecule/context.	Low for the single-variable assumption	Long-read transcript-origin mapping and isoform-specific protein/transcript assays.
IGF2-high HBV-HCC is a universal therapeutic target	The axis is better framed as a biomarker-development hypothesis in a subset of HBV-related HCC.	These findings support anti-IGF treatment in HBV-HCC.	Low for therapy claim	Prospective subset definition and biomarker-enriched trials.

**Table 6:** Clinical biomarker intended-use scenarios for an HBx-IGF2 axis in HBV-related HCC.

Use Case	Population/Sample	Candidate Assay	Comparator	Endpoint	Current Evidence	Required Validation	Actionability
Post-resection recurrence risk stratification	HBV-related resected HCC; tumor tissue ± adjacent liver	Promoter-resolved P3/P4:P1 qRT-PCR/RNA-seq; targeted methylation assay	AFP, tumor size, microvascular invasion, Edmondson grade	RFS/DFS	Indirectly supported by P3/P4 association with aggressive phenotype; no validated assay.	Multicenter retrospective training/validation with predefined cutoffs.	Research only
Aggressive pathology annotation	HBV-related surgical HCC; tumor tissue	P4 methylation; P3/P4 transcript assay	Routine histopathology	Poor differentiation, PVTT, vascular invasion	Human association data exist for P4- and P3-related aggressiveness.	Independent cohorts and multivariable clinicopathologic models.	Research only
Molecular subclassing in translational studies	HBV-related HCC biobanks	P3/P4 expression + methylation + HBx source/form + integration mapping	Etiology-only grouping	Biological subset definition	Conceptually strong, but not standardized.	Harmonized assay panel and cross-platform concordance.	High research utility; no clinical use
Prognostic stratification under current systemic therapy	Advanced/unresectable HBV-HCC receiving ICI-based therapy; pretreatment tissue ± exploratory plasma	Tissue RNA/methylation panel with integration-aware sequencing	BCLC stage, AFP, liver function, treatment regimen	OS, PFS, ORR, durable benefit	Essentially absent in current literature.	Prospective biomarker cohort in the modern treatment era.	Not actionable yet
Predictive enrichment for pathway-directed combinations	Molecularly selected HBV-HCC subset	Composite fetal-IGF2 locus signature	Unselected enrollment	Response to IGF-axis or rational combination therapy	No validated predictive evidence.	Analytical validity, clinical validity, then enrichment trial.	Do not use clinically
Field-effect or surveillance adjunct in chronic HBV	Advanced fibrosis/cirrhosis or suppressed/cured HBV populations; tissue or liquid biopsy exploratory	Methylation-based assay; cfRNA/cfDNA exploratory	Standard HCC surveillance	Incident HCC	Unsupported at present.	Longitudinal surveillance cohorts and pre-analytic standardization.	Hypothesis only

Technical practicality: For retrospective FFPE-based clinical cohorts, targeted P3/P4:P1 qRT-PCR or NanoString assays combined with targeted P3/P4 methylation testing are more immediately deployable than long-read transcriptomics, phased methylome analysis, or chromosome-conformation assays. Long-read, phased methylome, and Capture-C/Hi-C approaches are better suited to smaller mechanistic discovery cohorts before translation into scalable biomarker panels.

**Table 7:** Priority research agenda for promoter-resolved, source-aware HBx-IGF2 studies.

Priority Question	Required Design	Minimum Readouts	Expected Decision Value
Does endogenous HBx activate IGF2 P4 or P3/P4 in natural infection systems?	HBV infection-competent systems with HBx-deficient/rescue controls.	Promoter-resolved RNA, total IGF2/IGF-II, HBx abundance, cccDNA transcription.	Separates reporter-derived mechanisms from endogenous locus behavior.
Are P3/P4 methylation changes allele-specific and linked to LOI?	Human tissues or organoids with informative SNPs and phased methylome.	P3/P4 methylation, allele-specific expression, promoter usage.	Prevents conflation of promoter switching, hypomethylation, and LOI.
Does HBx directly alter IGF2/H19 topology?	Source-aware HBx perturbation with Capture-C/4C/Hi-C and CTCF/cohesin profiling.	Looping, enhancer contact, CTCF/cohesin occupancy, P3/P4 expression.	Tests the currently unproven domain-level claim.
Do HBx forms differ in IGF2 regulation?	Isoform-specific full-length/truncated/fusion HBx models and clinical annotation.	HBx isoform, subcellular localization, P3/P4 expression, methylation, histone acetylation.	Determines whether tumor-stage HBx can be treated as one variable.
Does the fetal-IGF2 locus state have clinical utility?	Prospective HBV-HCC cohorts under contemporary ICI/TKI therapy.	P3/P4:P1 ratio, methylation, integration mapping, AFP, stage, liver function, outcomes.	Determines incremental prognostic or predictive value.
Can HBx source and form be linked to promoter-specific IGF2 activity in patient tissue?	Paired tumor and adjacent liver tissue from HBV-related HCC, analyzed by long-read viral transcriptome sequencing or integration-aware DNA/RNA sequencing, with isoform-specific HBx annotation in the same specimens.	Episomal versus integrated viral transcript origin; full-length, C-terminally truncated, and fusion-derived HBx; IGF2 P3/P4:P1 transcript ratio; P3/P4 methylation; selected H3/H4 acetylation or HBx-associated chromatin readouts when tissue permits.	Converts source-aware framing into a testable tissue-level design and prevents tumor-stage HBx from being treated as a single biological variable.

## Data Availability

No new data were created or analyzed in this study. Data sharing is not applicable to this article.
